# Phytochemical Investigation of *Stachys hissarica*: Antioxidant Activity, Flavonoid Characterization, and In Silico Analysis

**DOI:** 10.1002/cbdv.71813

**Published:** 2026-10-15

**Authors:** Elbek A. Mukhammadiev, Michal Šoral, Francesco Cacciola, Katia Arena, Chao Liu, Mengqi Zhang, Ruirui Zhang, Doniyor R. Siddikov, Sobirdjan A. Sasmakov, Nilufar Z. Mamadalieva

**Affiliations:** ^1^ Institute of the Chemistry of Plant Substances Uzbekistan Academy of Sciences Tashkent Uzbekistan; ^2^ Analytical Department, Institute of Chemistry Slovak Academy of Sciences Bratislava Slovak Republic; ^3^ Messina Institute of Technology, c/o Department of Chemical, Biological, Pharmaceutical and Environmental Sciences, Former Veterinary School University of Messina Messina Italy; ^4^ Key Laboratory of Novel Food Resources Processing, Institute of Food & Nutrition Science and Technology, Ministry of Agriculture and Rural Affairs Shandong Academy of Agricultural Sciences Jinan China; ^5^ Tea Research Institute Shandong Academy of Agricultural Sciences Jinan China; ^6^ Faculty of Medicine Alfraganus University Tashkent Uzbekistan

**Keywords:** antioxidant, ESI‐MS/MS, *Stachys hissarica*, flavonoids, NMR, phenolics

## Abstract

Evaluating *Stachys hissarica* extracts via DPPH, FRAP, and ABTS assays revealed that the aqueous methanolic fraction exhibited strong, ascorbic acid‐comparable antioxidant activity. This high potency confirms that hydrophilic phenolic compounds are the primary drivers of its radical‐scavenging effect. HPLC‐PDA/ESI‐MS/MS analysis of the aqueous methanolic extract led to the tentative annotation of 35 metabolites, including hydroxycinnamic acid derivatives, flavonoid glycosides, acetylated and methoxylated flavones, consistent with the phytochemical profile of the *Stachys* genus. The antioxidant activities of these polyphenolic constituents likely support the strong radical‐scavenging activity observed. In addition, five compounds were isolated from the polar extract of *S. hissarica*, and their chemical structures were elucidated using NMR and high‐resolution mass spectrometry. Computer simulations showed that hydrophilic phenolics, such as naringenin and hesperidin, possess favorable oral bioavailability, low toxicity, and strong, stable binding affinities to the target protein. However, these computational predictions require experimental validation through biochemical and pharmacological assays.

## Graphical abstract

*Stachys hissarica* demonstrates remarkable antioxidant potential, driven by a rich profile of 35 annotated polyphenolic metabolites within its aqueous methanolic extract. Hydrophilic phenolics exhibit superior radical‐scavenging activity comparable to ascorbic acid. Integrated computational simulations reveal favorable oral bioavailability, low toxicity, and strong target‐binding affinities, underscoring this endemic plant as a promising source for natural therapeutic antioxidants.

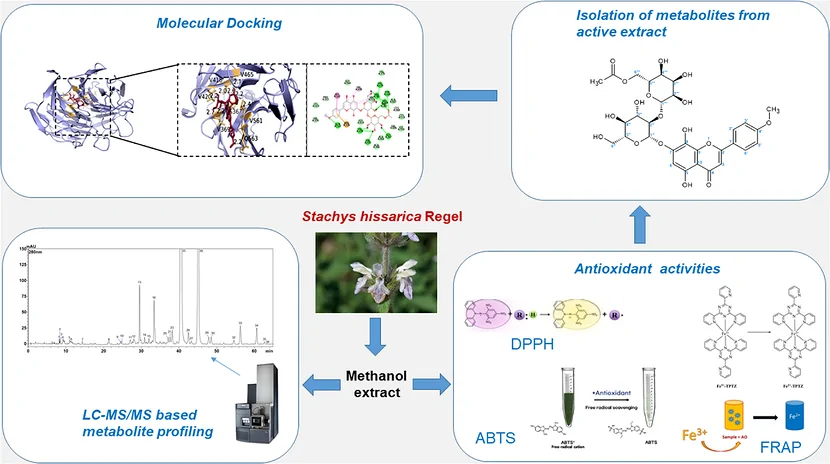

## 1 Introduction

The genus *Stachys* L., a member of the Lamiaceae family, comprises approximately 300 annual or perennial herbaceous plants and small shrubs, which are widely distributed in temperate and tropical regions across Europe, Asia, the Americas, and northern and southern Africa [1, 2]. *Stachys* species have a long history of use in traditional medicine, reflecting their broad therapeutic spectrum. In Mediterranean and Middle Eastern folk practices, *Stachys* species are used to treat gastrointestinal disorders, respiratory infections, wounds, rheumatism, gynecological problems, and psychological conditions, such as anxiety and depression. For example, *S. lavandulifolia* is well‐known in Iranian traditional medicine as a remedy for anxiety, insomnia, and gastrointestinal discomfort and is commonly consumed as an herbal tea called “Chaaye Koohi” [1, 2]. Recent studies have demonstrated notable cytotoxic and antioxidant activities in various extracts derived from different *Stachys* species, including *S. aegyptica*, *S. parviflora*, *S. affinis*, *S. lavandulifolia*, and *S. cretica* [3].

To date, more than 250 distinct compounds have been isolated from various *Stachys* species, primarily from their aerial parts. Phytochemical investigations have revealed that members of this genus are rich in diverse bioactive compounds, including flavonoids, terpenoids (triterpenes, diterpenes, and iridoids), polyphenols (flavone derivatives, phenylethanoid glucosides, and lignans), phenolic acids, and essential oils [1, 2]. Among these constituents, flavonoids are particularly characteristic of the genus and occur mainly as flavones, polymethylated flavones, flavonols, flavanones, and their glycosides. Frequently reported compounds include apigenin, luteolin, kaempferol, quercetin, isorhamnetin, naringenin, hesperidin, and various glycosylated and acylated derivatives [4]. Phenylethanoid glycosides, such as acteoside, isoacteoside, forsythoside B, and leucosceptoside B, are also widely distributed among *Stachys* species, while hydroxycinnamic acid derivatives, particularly caffeic and chlorogenic acids, contribute substantially to the phenolic profile of several species [4, 5, 6].

The complex chemical profiles of these plants underpin their diverse pharmacological properties, including antioxidant, anti‐inflammatory, antimicrobial, analgesic, cytotoxic, renoprotective, and neuroactive effects [7, 8, 9]. Essential oils derived from *Stachys* species further contribute to their pharmacological profile, particularly through antimicrobial and anti‐inflammatory actions. Several species, such as *S. aegyptica*, *S. lavandulifolia*, *S. affinis*, and *S. cretica* have demonstrated potent free radical scavenging activity and inhibitory effects on cancer cell proliferation in vitro [1, 2]. Studies have shown that extracts of *Stachys* species can effectively scavenge free radicals and exhibit reducing and lipid‐peroxidation‐inhibitory activities, as demonstrated by assays such as DPPH (1,1‐diphenyl‐2‐picrylhydrazyl radical scavenging), FRAP (ferric reducing antioxidant power), ABTS (2,2‐azino bis (3‐ethylbenzothiazoline‐6‐sulfonic acid)), hydroxyl‐radical scavenging, and lipid peroxidation tests. In a comparative study of six *Stachys* species, *S. germanica*, *S. byzantina*, and *S. palustris* exhibited particularly strong antiradical activity, which was associated with their relatively high total phenolic, flavonoid, and caffeic‐acid‐derivative contents; chlorogenic acid was identified as the predominant phenolic compound [5]. Similarly, Kukić et al. reported considerable antioxidant activity in several *Stachys* species, including *S. anisochila*, *S. beckeana*, *S. plumosa*, and *S. alpina subsp. dinarica* [10]. Among these taxa, *S. beckeana* exhibited 64.97% hydroxyl‐radical scavenging activity, while the antioxidant effects of the extracts were strongly correlated with their total phenolic contents. These findings further support the potential of *Stachys* species as important natural sources of antioxidant compounds.

Despite the increasing number of phytochemical and pharmacological studies, many *Stachys* species remain underexplored, especially those endemic to Central Asia. Five *Stachys* species have been confirmed to occur in Uzbekistan by recognized plant databases (https://powo.science.kew.org). *S. hissarica* Regel (local name: Xisor tog’ quddusi) is commonly used in traditional medicine to treat wound healing and gastrointestinal disorders and respiratory infections. The plant reaches a height of 40–70 cm and grows on stony slopes in the middle mountain belt. Its general distribution includes Central Asia, specifically the Western Tien Shan and Pamir‐Alay ranges. In Uzbekistan, it is found in the mountainous regions of Tashkent, Samarkand, and Surkhandarya. Flowering occurs from June to August, and fruiting from July to September [11].

Despite its traditional use in folk medicine, the pharmacological properties and phytochemical composition of *S. hissarica* remain insufficiently investigated. To date, phytochemical studies of this species have been limited, with previous investigations mainly focusing on its essential oil composition and, more recently, the reported occurrence of ecdysteroids [12, 13]. In this context, the present study aimed to comprehensively characterize the secondary metabolites of the aqueous methanolic extract of *S. hissarica* using UHPLC‐ESI‐MS/MS and to isolate and structurally elucidate selected flavonoid constituents by high‐resolution mass spectrometry and NMR spectroscopy. The antioxidant potential of the extract and its fractions was also evaluated using complementary in vitro assays, including DPPH, FRAP, and ABTS. In addition, representative phenolic compounds identified in the extract were subjected to in silico ADMET prediction and molecular docking analyses to explore their predicted pharmacokinetic properties, toxicity profiles, and interactions with selected molecular targets.

## 2 Results and Discussion

### 2.1 Antioxidant Activity of the Extracts of *S. hissarica*

In our previous investigation, the antioxidant potential of essential oils isolated from three *Stachys* species was investigated [12]. In this regard, different extracts of *S. hissarica* have been evaluated using antioxidant methods, such as DPPH, FRAP, and ABTS assays. The results of the antioxidant activity evaluation of various extracts of *S. hissarica* are summarized in Table 1.

**TABLE 1:** Antioxidant activity of *S. hissarica* extracts.

Extract or standard	DPPH	FRAP	ABTS
	IC_50_, µg/mL		
MeOH	54.23 ± 1.40	379.21 ± 11.07	14.35 ± 0.93
CHCl_3_	142.81 ± 8.65	986.42 ± 54.21	62.56 ± 3.21
EtOAc	78.43 ± 3.20	287.45 ± 13.97	7.42 ± 0.43
MeOH:H_2_O (70:30, v/v)	12.32 ± 0.58	201.86 ± 15.53	3.23 ± 0.21
BuOH	61.03 ± 4.89	476.31 ± 29.42	23.46 ± 1.89
Ascorbic acid	22.21 ± 2.01	n.d.	4.35 ± 0.41
Trolox	n.d.	38.21 ± 1.19	n.d.

*Note*: Values are reported as mean ± S.D. of three parallel measurements.

Abbreviations: IC_50_, half inhibitory concentration; n.d., not determined.

The antioxidant potential of different *S. hissarica* extracts was assessed using DPPH, FRAP, and ABTS assays, revealing clear polarity‐dependent differences. The aqueous methanolic extract exhibited the strongest radical‐scavenging activity, with IC_50_ values of 12.32 ± 0.58 µg/mL (DPPH) and 3.23 ± 0.21 µg/mL (ABTS), comparable to ascorbic acid (22.21 ± 2.01 µg/mL and 4.35 ± 0.41 µg/mL, respectively), and a FRAP value of 201.86 ± 15.53 µg/mL. In contrast, the CHCl_3_ and EtOAc extracts showed considerably weaker radical‐scavenging capacities, with IC_50_ values of 142.81 ± 8.65 µg/mL and 78.43 ± 3.20 µg/mL in the DPPH assay, respectively. The CHCl_3_ extract exhibited the highest FRAP value (986.42 ± 54.21 µg/mL), indicating substantial ferric‐reducing power despite its weaker DPPH and ABTS activities. This suggests that the CHCl_3_ fraction may contain non‐radical‐scavenging, electron‐donating compounds. The BuOH and MeOH extracts exhibited moderate antioxidant activities. The BuOH extract had an IC_50_ of 61.03 ± 4.89 µg/mL in the DPPH assay and an ABTS value of 23.46 ± 1.89 µg/mL, while the MeOH extract demonstrated slightly better DPPH scavenging (IC_50_ = 54.23 ± 1.40 µg/mL) and similar ferric‐reducing power (379.21 ± 11.07 µg/mL) compared to BuOH.

The findings indicated that polar extracts (especially aqueous methanolic) exhibited stronger free radical scavenging capacity, suggesting the presence of hydrophilic phenolic or polyphenolic compounds. These results are consistent with previous reports highlighting the high antioxidant potential of polar fractions from *Stachys* species, especially aqueous methanol, which is particularly enriched in phenolic constituents responsible for strong antioxidant capacity [14]. Notably, our earlier study on *S. hissarica* essential oil also demonstrated high antioxidant activity [12]. Among the tested samples, only *S. hissarica* essential oil exhibited strong activities, particularly in the antioxidant assays (13.04 mg TE/g oil for DPPH, 109.55 mg TE/g oil for FRAP, and 226.48 mg TE/g oil for ABTS), further supporting the bioactivity of this species across different extract types. Nevertheless, further cellular and in vivo studies are required to validate the pharmacological and therapeutic potential of these compounds and to determine their biological relevance and efficacy under physiological conditions.

### 2.2 LC‐MS/MS Investigations

LC‐MS/MS analysis is essential for plant extract investigation as it enables highly sensitive, selective, and accurate identification and quantification of complex mixtures of bioactive metabolites [15, 16]. The results of the antioxidant activity evaluation of various *S. hissarica* extracts revealed that the aqueous methanolic extract exhibited the strongest antioxidant capacity. Therefore, we proceeded to identify the metabolites present in this extract using the HPLC‐MS method (Figure S1). LC‐PDA/ESI‐MS/MS analysis of the MeOH:H_2_O (70:30, v/v) extract of *S. hissarica* led to the tentative identification of 35 metabolites (Table 2). These compounds mainly belonged to the classes of hydroxycinnamic acid derivatives, flavonoid glycosides, and methoxylated flavones, consistent with the previously reported chemical composition of the *Stachys* genus [1, 2, 17]. The annotation of these compounds was based on retention times (*t*_R_), UV absorption maxima (*λ*_max_), accurate mass data ([M‐H]^−^/[M+H]^+^), and fragmentation patterns (MS/MS), with reference to previously reported metabolites from related *Stachys* species.

**TABLE 2:** Identification of phenolic compounds in MeOH:H_2_O (70:30, *v/v*) extracts of *S. hissarica* through LC‐PDA/ESI‐MS/MS analysis (*λ* = 280 nm)^a^.

Nos.	Compounds	*t*_R_ (min)	Molecular formulae	Mr	*λ*_max_, nm	[M−H]^−^	[M+H]^+^	MS/MS	Other source organisms reported in the literature	References
**1**	Caffeoylrutinoside isomer	8.20	C_27_H_30_O_15_	594.52	245, 328	593		325^+^[Rutinose]; 181^+^[caffeic acid]	*S. recta*; *S. palustris*	[1]
**2**	Caffeoylrutinoside isomer	8.37	C_27_H_30_O_15_	594.52	245, 329	593		325^+^[Rutinose]; 181^+^[caffeic acid]	*S. recta*; *S. palustris*	[1]
**3**	Unknown	8.46	—		265, 343	361	363			
**4**	Unknown	9.20	—		279	333	335	121^+^		
**5**	Feruloylrutinoside	11.00	C_28_H_32_O_15_	608.55	246, 327	607		325^+^ [Rutinose]; 195^+^ [ferulic acid]		
**6**	Unknown	11.37	—		281	363	365	151^+^; 123^+^		
**7**	Methoxyeugenol	14.32	C_11_H_14_O_3_	194.23	283	193	195	121^+^		
**8**	Hydroxycinnamic acid derivatives	21.40	—		315	331		255^+^; 197^+^; 183^+^		
**9**	Naringenin dihexoside isomer	24.48	C_27_H_32_O_14_	580.54	283, 337sh	579		271^−^[Naringenin]		
**10**	Naringenin dihexoside isomer	24.83	C_27_H_32_O_14_	580.54	283, 337sh	579		271^−^[Naringenin]		
**11**	Caffeic acid derivative	27.13	—		329	653		179^−^[Caffeic acid]		
**12**	Hydroxycinnamic acid derivative	27.99	—		326	361	331	269^+^; 243^+^; 171^+^		
**13** ^b^	Isoscutellarein‐7‐*O*‐[allopyranosyl‐(1→2)]‐glucopyranoside	29.52	C_27_H_30_O_16_	610.52	276, 304, 326	609	611	287^+^[Isoscutellarein]	*S. aegyptiaca*, *S. ocymastrum*, *S. subnuda*, *S. recta*, and *S. anisochila*	[1, 17]
**14**	Hydroxycinnamic acid derivative	30.87	—		325	431	433			
**15**	Isoscutellarein‐7‐*O*‐acetylated‐dihexoside isomer	31.99	C_29_H_32_O_17_	652.56	276, 305, 326	651	653	287^+^[Isoscutellarein]		[17]
**16**	Tetrahydroxymethoxyflavone‐dihexoside	33.37	C_28_H_32_O_17_	640.55	276, 338	639	641	317^+^[M‐Dihexose]		
**17**	Trihydroxyflavanone‐hexuronide‐acetylhexoside	34.95	C_29_H_32_O_17_	652.56	278, 325sh	651		447^−^[M‐Acetylhexose]; 271^−^[trihydroxyflavanone]		
**18**	Trihydroxyflavanone‐hexoside‐acetylhexoside	35.58	C_29_H_34_O_16_	638.57	283, 331sh	637	639	595^−^[M‐Acetyl]; 475^−^[M‐hexose]; 271^−^[trihydroxyflavanone]		[17]
**19**	3′‐Hydroxy‐4′‐*O*‐methylisoscutellarein‐7‐*O*‐acetylated dihexoside isomer	36.14	C_30_H_34_O_18_	682.58	276, 343	681	683	317^+^[Hydroxymethylisoscutellarein]; 301^+^[hydroxymethylisoscutellarein‐methyl]		[17]
**20**	Isoscutellarein‐7‐*O*‐acetylated‐dihexoside isomer	37.05	C_29_H_32_O_17_	652.56	276, 305, 326	651	653	449^+^[M‐Acetylhexose]; 287^+^[isoscutellarein]		[17]
**21**	Isoscutellarein‐glucoside	37.61	C_21_H_20_O_11_	448.38	278, 304, 329	447	449	287^+^[Isoscutellarein]	*S. aegyptiaca*	[20]
**22**	Apigenin acetyl‐allopyranosyl‐glucoside	38.04	C_29_H_32_O_16_	636.56	267, 337	635	637	271^+^[Apigenin]	*S. palustris* and *S. aegyptiaca*	[21]
**23**	Isoscutellarein‐7‐*O*‐acetylated‐dihexoside isomer	38.22	C_29_H_32_O_17_	652.56	276, 305, 326	651	653	287^+^[Isoscutellarein]		[17]
**24**	3′‐Hydroxy‐4′‐*O*‐methylisoscutellarein‐7‐*O*‐acetylated‐dihexoside isomer	39.39	C_30_H_34_O_18_	682.58	274, 343	681	683	317^+^[Hydroxymethylisoscutellarein]; 301^+^[hydroxymethylisoscutellarein‐methyl]		[17]
**25**	Isoscutellarein‐7‐*O*‐acetylated‐dihexoside isomer	40.49	C_29_H_32_O_17_	652.56	276, 305, 326	651	653	287^+^ [Isoscutellarein]	*S. aegyptiaca*, *S. candida*, *S. iva*, *S. corsica*, *S. alpine*, *S. germanica*, *S. lanata*, *S. tymphaea*, *S. subnuda*, *S. atherocalyx*, *S. angustifolia*, *S. annua*, *S. parolinii*, *S. recta*, *S. labiosa*, *S. subcrenata*, *S. baldaccii*, *S. tetragona*, *S. anisochila*, *S. menthifolia*, *S. ionica*, *S. sieboldii*, *S. mialhesii*, and *S. subnuda*	[1, 17, 19]
**26**	3′‐Hydroxy‐4′‐*O*‐methylisoscutellarein‐7‐*O*‐acetylated‐dihexoside isomer	42.46	C_30_H_34_O_18_	682.58	271, 343	681	683	477^−^[M‐Acetylhexose]; 315^−^[hydroxymethylisoscutellarein]		
**27**	Tetrahydroxymethoxyflavone isomer	43.30	C_16_H_12_O_7_	316.26	278, 337	315	317	302^+^[M‐Methyl]		
**28** ^b^	4′‐*O*‐Methylhypolaetin 7‐*O*‐[6‴‐*O*‐acetyl‐β‐D‐allopyranosyl(1→2)‐β‐D‐glucopyranoside] (syn. 3′‐hydroxy‐4′‐*O*‐methylisoscutellarein 7‐*O*‐[6‴‐*O*‐acetyl‐β‐D‐allopyranosyl‐(1→2)‐β‐D‐glucopyranoside])	45.14	C_30_H_34_O_18_	682.58	276, 340	681	683	317^+^; 302^+^		[17]
**29**	Tetrahydroxymethoxyflavone isomer	47.82	C_16_H_12_O_7_	316.26	278, 337	315	317	302^+^[M‐Methyl]		
**30**	Leonoside B	48.53	C_36_H_48_O_19_	784.76	329	783		651^−^[M‐Pentose]; 475^−^[M‐pentose‐ferulic acid]; 193^−^[ferulic acid]	*S. lanata*	[22]
**31**	Isoscutellarein‐7‐*O*‐acetylated‐dihexoside isomer	52.55	C_29_H_32_O_17_	652.56	278, 330	651	653	491^+^[M‐Hexose]; 287^+^[isoscutellarein]		
**32**	Trihydroxydimethoxyflavone isomer	54.57	C_16_H_12_O_6_	300.26	283, 336	299	301	286^+^[M‐Methyl]	*S. aegyptiaca*	[20]
**33**	Trihydroxymethoxyflavone isomer	56.27	C_16_H_12_O_6_	300.26	283, 336	299	301	286^+^[M‐Methyl]		
**34**	Trihydroxydimethoxyflavone	60.60	C_17_H_14_O_7_	330.29	278, 337	329	331	316^+^[M‐Methyl]; 301^+^[M‐methyl‐methyl]		
**35**	Tetramethoxyflavanone‐acetylated‐dihexoside isomer	62.70	C_33_H_42_O_17_	686.67	281, 321	685	687	645^+^[M‐Acetyl]; 483^+^[M‐acetylhexose]		

^a^Compounds tentatively annotated based on MS/MS data and data from the literature of natural compounds observed in the *Stachys* genus.

^b^Compounds confirmed with authentic reference standard.

Several peaks (**1**, **2**, **5**, and **11**) were assigned to caffeoyl‐ and feruloyl‐*O*‐rutinose esters based on their characteristic fragment ions. These were observed at *m/z* 181 or 179 ([M+H]^+^ or [M‐H]^−^ of caffeic acid) for derivatives **1**, **2**, and **11**, and at *m/z* 193 ([M‐H]^−^ of ferulic acid) for feruloylrutinoside (**5**). The fragmentation spectra of compounds **1** and **2** also contained the diagnostic signal *m/z* 325 [M+H‐hexose]^−^. Aglycones of these phenolic compounds have been described in *S. recta, S. palustris* and other species, and are known for their potent antioxidant properties [5], this supports the strong radical scavenging activity observed in this extract.

Flavonoid glycosides, particularly isoscutellarein derivatives, were among the most abundant metabolites in the extract. Isoscutellarein‐7‐*O*‐[allopyranosyl‐(1→2)]‐glucopyranoside (**13**, *m/z* 609 [M‐H]^−^) was detected in the water fraction and has been reported in *S. aegyptiaca*, *S. ocymastrum*, *S. subnuda*, *S. recta*, and *S. anisochila* [17, 18]. Several acetylated derivatives (compounds **15**, **20**, **23**, **25**, and **31**) showed diagnostic fragment ions at *m/z* 287, belonging to [M+H]^+^ of isoscutellarein. These compounds exhibited strong UV absorbance in the 276–326 nm range, characteristic of flavones, and have been previously reported in related *Stachys* species [5]. In particular, compound **25** (isoscutellarein‐7‐*O*‐acetylated‐dihexoside) was putatively annotated as a major metabolite, which has been widely reported in numerous *Stachys* species, including *S. aegyptiaca*, *S. candida*, *S. iva*, *S. corsica*, *S. germanica*, and *S. lanata* [1, 17, 19]. This observation highlights the chemotaxonomic value of acetylated isoscutellarein glycosides within the genus. Isoscutellarein glucoside (**21**, *m/z* 443 [M–H]^−^ and *m/z* 445 [M+H]^+^) was also detected, consistent with its occurrence in *S. aegyptiaca* [20]. In addition, peak **22** was tentatively identified as apigenin acetyl‐allosyl‐glucoside based on its fragmentation ion at *m/z* 271, which is indicative of the apigenin core ([M+H]^+^ of apigenin). This compound has previously been described in *S. palustris* and *S. aegyptiaca* [21]. Among the most abundant flavonoid glycosides tentatively identified were naringenin dihexosides (**9** and **10**), with prominent fragment ions at *m/z* 271 [naringenin aglycone‐H]^−^, in agreement with findings from related *Stachys* species [17]. These flavonoid glycosides are known antioxidants and are likely key contributors to the strong radical scavenging observed in the assays.

A series of methylated and acetylated flavones and flavanones were also putatively annotated in the later eluting region, such as trihydroxy‐ and tetrahydroxymethoxyflavones (**17**, **18**, **27**, **29**, and **32–35**) and 3′‐hydroxy‐4′‐*O*‐methylisoscutellarein derivatives (**19**, **24**, **26**, and **28**). These compounds featured key MS/MS fragments (*m/z* 317, 301, and 286), indicating losses of methyl or acetyl groups. Structural modifications, such as methylation and acetylation, observed in these flavones, may enhance lipophilicity and biological activity [4]. In addition, leonoside B (**30**) was detected with a molecular ion at *m/z* 783 [M‐H]^−^, along with fragments corresponding to pentose and ferulic acid losses. This phenylethanoid glycoside, previously reported in *S. lanata* [22] reinforces the structural similarity of *S. hissarica* with other members of the genus and is recognized for its potent antioxidant properties. Several unidentified compounds (**3**, **4**, and **6**) with distinct UV absorption and MS/MS fragmentation patterns were also detected, indicating the presence of potentially novel metabolites.

These unique constituents may represent new chemotypes of *S. hissarica* and warrant future isolation and characterization. Taken together, the antioxidant assay results and LC‐MS/MS annotation strongly suggest that the high radical‐scavenging activity of *S. hissarica*, particularly in the aqueous methanolic fraction, is a result of the antioxidant action of hydroxycinnamic acid derivatives, isoscutellarein glycosides, flavones, and phenylethanoid glycosides. This compositional complexity, dominated by phenolic and flavonoid glycosides, provides a solid explanation for the pronounced antioxidant activity observed in the aqueous methanolic extract. The presence of compounds previously reported in numerous *Stachys* species confirms the chemotaxonomic consistency of the genus, whereas the detection of unknown metabolites highlights the potential of *S. hissarica* as a promising source of novel bioactive phytochemicals.

### 2.3 Identification of Isolated Compounds

The Unbiased by the results of the LC‐MS/MS analysis described above, NMR spectroscopy‐based identification of selected isolated compounds was attempted. After the isolation of several compounds in sufficient amount and purity (the methodology is described in the Section 4.5), their NMR samples were prepared and subjected to measurement. The NMR analysis resulted in the identification of naringenin (**36**), hesperetin (**37**), isoscutellarein 7‐*O*‐[β‐D‐allopyranosyl(1→2)‐β‐D‐glucopyranoside] (**13**), 4′‐*O*‐methylisoscutellarein 7‐*O*‐[6‴‐*O*‐acetyl‐β‐D‐allopyranosyl(1→2)‐β‐D‐glucopyranoside] (**38**), and 4′‐*O*‐methylhypolaetin 7‐*O*‐[6‴‐*O*‐acetyl‐β‐D‐allopyranosyl(1→2)‐β‐D‐glucopyranoside] (syn. 3′‐hydroxy‐4′‐*O*‐methylisoscutellarein 7‐*O*‐[6‴‐*O*‐acetyl‐β‐D‐allopyranosyl‐(1→2)‐β‐D‐glucopyranoside]) (**28**) (Figure 1) (NMR signal assignments can be found in Tables S1–S5 and copies of acquired NMR spectra are depicted in Figures S2–S29).

**FIGURE 1:**
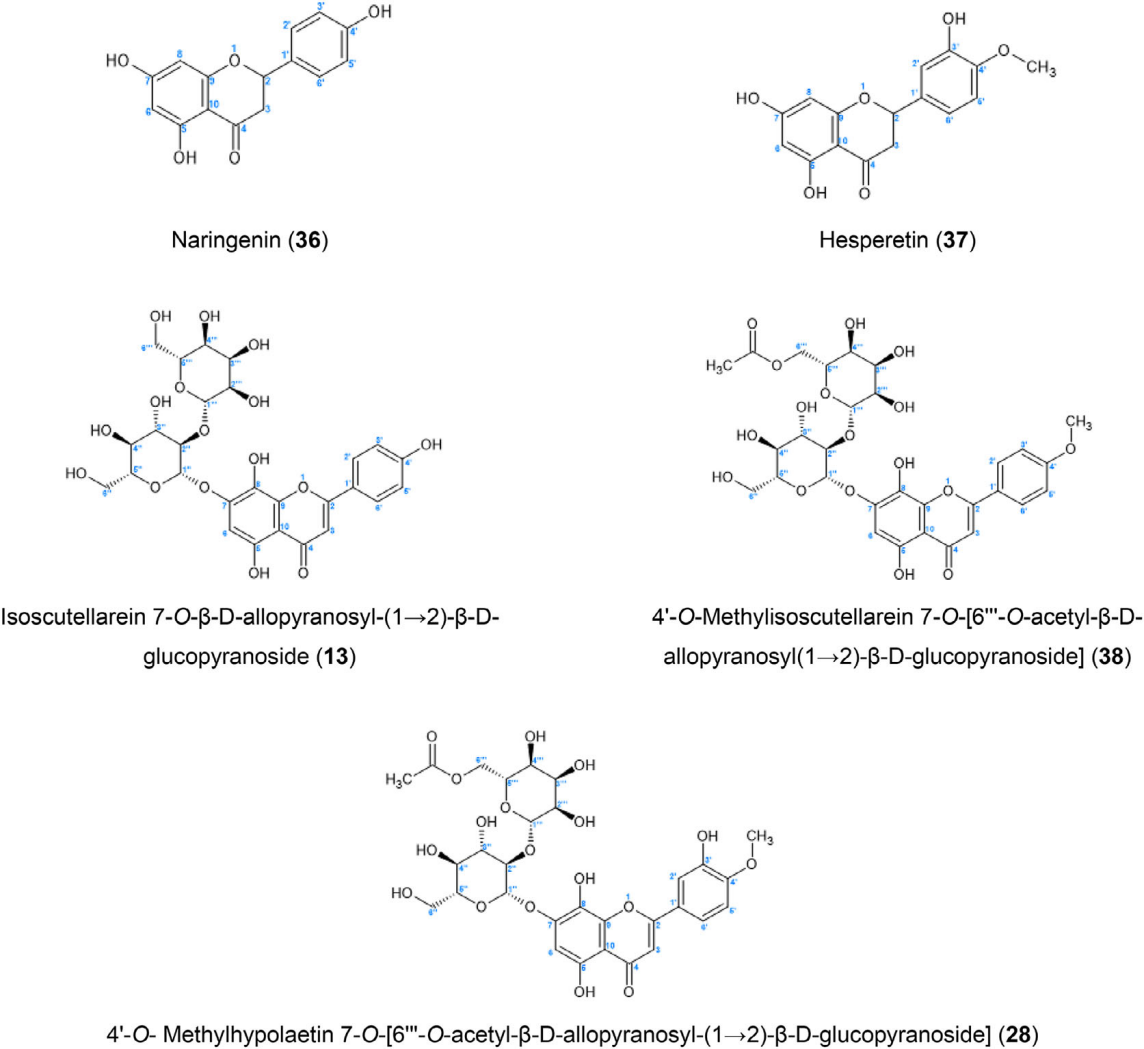
Isolated compounds **13, 28**, and **36–38** from *S. hissarica*.

The identification was based on the analyses of the compounds’ 1D and 2D NMR spectra—the analyses provided sufficient data for the elucidation of the compounds’ structures with good reliability. The only ambiguity in the structures was the possibly wrong identification of the substitution on the A‐ring of the glycosylated compounds.

The three series of ^1^H and ^13^C NMR chemical shifts originating from the A‐ring of the aglycons of the 3 glycosylated compounds (obtained from their pyridine‐*d*_5_ solutions) were very similar, but only the relative positions of the proton and hydroxy‐groups of the A‐ring could be obtained—especially the positions 6 and 8 could be mutually swapped. This was caused by the inability to obtain HMBC correlations from the 5‐OH protons due to their signal being broadened by chemical exchange. For this reason, 4′‐*O*‐methylisoscutellarein 7‐*O*‐[6‴‐*O*‐acetyl‐β‐D‐allopyranosyl(1→2)‐β‐D‐glucopyranoside] (**38**) was dissolved in DMSO‐*d*_6_ with two intentions—to correlate the NMR data with available NMR data in literature [23], and to possibly obtain enough correlation data to unambiguously identify the structure.

Indeed, the ^1^H NMR spectrum of **38** dissolved in DMSO‐*d*_6_ provided a sharp 5‐OH signal (identified from its high *δ*_H_ value resulting from a hydrogen bond with the carbonyl in the 4‐position) and its three HMBC correlations (Figure 2), including a correlation with the already‐assigned carbon C10; the NMR chemical shift values and signal assignments can be found in Table 3. After the unambiguous identification of the compound's structure, the NMR shifts were correlated with published data [23, 24, 25], and they were found to be in excellent agreement. Owing to the similarities of the NMR spectra (measured from their pyridine‐*d*_5_ solutions) of all isolated glycosylated compounds in our study, it was assumed that all of them are 7‐*O*‐glycosylated and have a hydroxyl group in the 8‐position.

**FIGURE 2:**
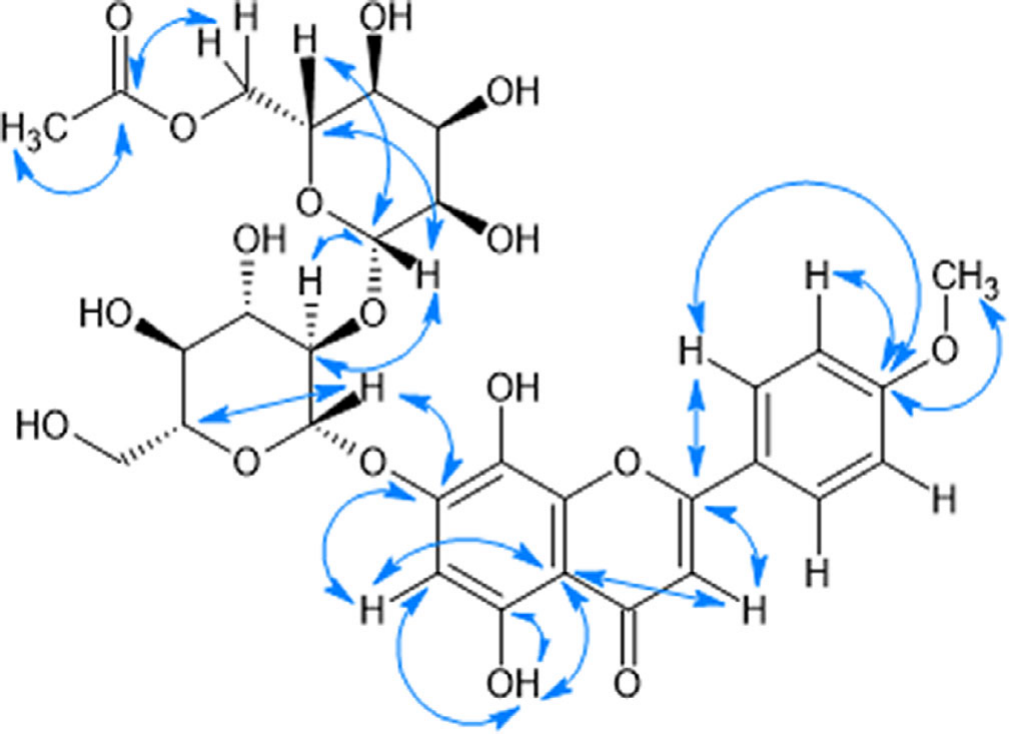
Selected HMBC correlations identified in the HMBC spectrum of 4′‐*O*‐methylisoscutellarein 7‐*O*‐[6‴‐*O*‐acetyl‐β‐D‐allopyranosyl(1→2)‐β‐D‐glucopyranoside] (**38**) dissolved in DMSO‐*d*_6_, that were pivotal in the identification of the compound's structure.

**TABLE 3:** The structure, atom notation and NMR signal assignment of 4′‐*O*‐methylisoscutellarein 7‐*O*‐[6‴‐*O*‐acetyl‐β‐D‐allopyranosyl(1→2)‐β‐D‐glucopyranoside] (**38**). The NMR data were obtained from a DMSO‐*d*_6_ solution of the compound; the spectra were measured at a 400 MHz frequency (for ^1^H nuclei) and 101 MHz frequency (for ^13^C nuclei).

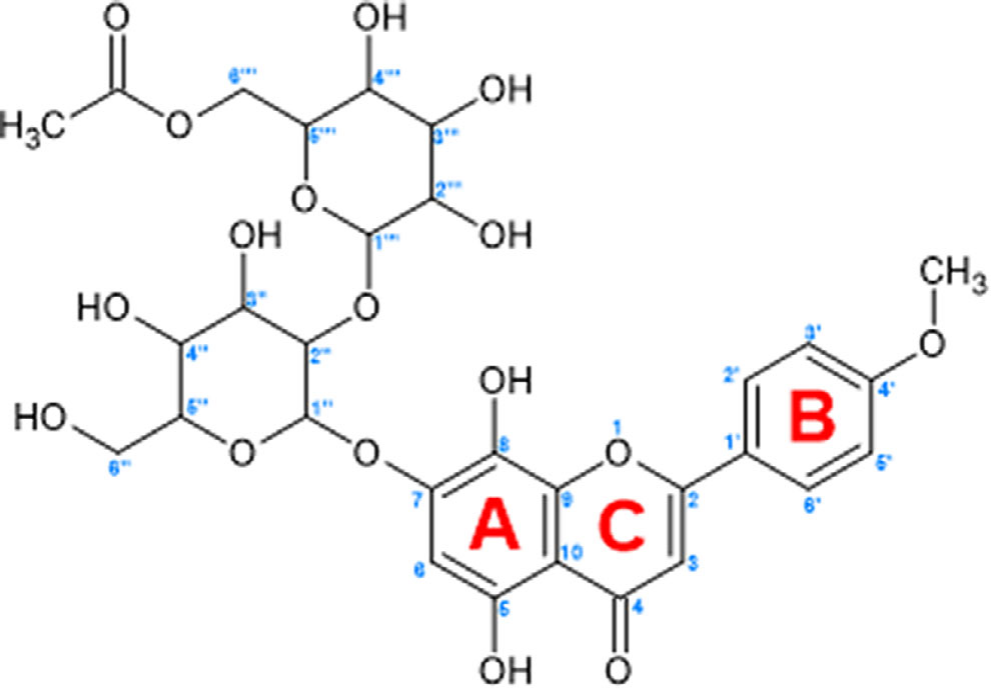				
Positions		^1^H chemical shift; *δ*_H_/ppm	Signal multiplicity; J_HH_/Hz	^13^C chemical shift; *δ*_C_/ppm
Ring C	1	—	—	—
	2	—	—	163.66
	3	6.924	s	103.37
	4	—	—	182.44
Ring A	5	—	—	152.22
	6	6.698	s	99.45
	7	—	—	150.64
	8	—	—	127.52
	9	—	—	143.79
	10	—	—	105.58
	5‐OH	12.315	s	—
	8‐OH	7.979	s	—
Ring B	1′	—	—	122.87
	2′	8.096	m	128.49
	3′	7.138	m	114.59
	4′	—	—	162.47
	5′	7.138	m	114.59
	6′	8.096	m	128.49
	4′‐OMe	3.871	s	55.59
β‐D‐glucopyranose	1″	5.077	d; 7.5	100.03
	2″	3.589	dd; 9.1, 7.5	82.52
	3″	3.509	t; 9.1	75.58
	4″	3.253	t, 9.1	69.24
	5″	3.467	m	77.14
	6″	3.495	m	60.54
		3.736	d; 10.9	
	3″‐OH	5.460	br s	—
	4″‐OH	5.213	br s	—
	6″‐OH	4.700	br s	—
β‐D‐allopyranose	1‴	4.922	d; 7.9	102.53
	2‴	3.250	dd; 7.9, 3.2	71.56
	3‴	3.913	t; 3.2	70.79
	4‴	3.402	m	66.87
	5‴	3.868	m	71.47
	6‴	4.008	dd; 11.9, 4.5	63.54
		4.040	dd; 11.9, 2.6	
	2‴‐OH	5.110	br s	—
	3‴‐OH	4.986	br s	—
	4‴‐OH	4.801	br s	—
	6‴‐OAc‐1	1.868	s	20.46
	6‴‐OAc‐2	—	—	170.31

An interesting aspect of the phytochemical profile of *S. hissarica* concerns the previously reported occurrence of ecdysteroids. Five ecdysteroids, including 20‐hydroxyecdysone, polypodin B, integristeron A, 2‐deoxy‐20‐hydroxyecdysone, and 2‐deoxyecdysone, were previously reported from this species [13]. However, the occurrence of these compounds appears to be unusual within the genus *Stachys*, as ecdysteroids have rarely been reported from *Stachys* species and the findings for *S. hissarica* originate from a single phytochemical investigation [1, 13]. Although the original study reported structural confirmation based on NMR, mass spectrometry, and IR spectroscopy, these findings have not, to our knowledge, been independently corroborated by subsequent phytochemical studies. Importantly, in the present investigation, despite comprehensive chromatographic and spectroscopic analyses of the polar extract, we found no evidence for the occurrence of the previously reported ecdysteroids. In contrast, our identification and isolation of flavonoid constituents provides additional evidence that phenolic metabolites represent an important component of the polar phytochemical profile of *S. hissarica*.

### 2.4 Molecular Docking

The aqueous methanolic extract of *S. hissarica* exhibited strong antioxidant activity in DPPH, FRAP, and ABTS assays, from which five flavonoid compounds (naringenin, hesperetin, and three glycosylated derivatives) were isolated. Flavonoids are well‐known natural antioxidants, and Keap1 has been reported in the literature as a key protein involved in the regulation of cellular antioxidant defense, with its Kelch domain serving as a binding platform for various flavonoids and natural polyphenols [26]. The Kelch domain features a conserved binding pocket composed of multiple valine and glycine residues, which is structurally suitable for accommodating flavonoid skeletons [27]. Therefore, Keap1 was selected as a model protein for in silico docking analysis to predict the potential binding modes and interactions of the isolated flavonoids with this antioxidant‐related target. The three‐dimensional structure of Keap1 (Kelch domain) was retrieved from the Protein Data Bank (PDB ID: 4IFJ) [28], which represents a high‐resolution x‐ray crystallographic structure at 1.80 Å resolution. The PDB entry 4IFJ has been validated with good Ramachandran statistics (0 outliers) [29], confirming the conformational reliability of the structure. Molecular docking experiments were performed using AutoDockTools 1.5.6 software to evaluate the binding affinity between target compounds and proteins [30]. The docking protocol was established following previously reported procedures for molecular docking against the Keap1 Kelch domain [31]. The docking results, including the binding energy and key interacting amino acids, are summarized in Table 4 and Figure 3.

**TABLE 4:** Binding affinity and interacting amino acids of compounds **13, 28**, and **36–38** from molecular docking.

Compounds	Binding energy (kcal/mol)	Binding amino acids
Naringenin (**36**)	−7.6	V561 and V369
Hesperetin (**37**)	−8.4	VAL‐418, VAL‐608, VAL‐561, and THR‐560
Isoscutellarein 7‐*O*‐β‐D‐allopyranosyl‐(1→2)‐β‐D‐glucopyranoside (**13**)	−9.5	V369, G367, V465, V324, and L559
4′‐*O*‐Methylisoscutellarein 7‐*O*‐[6‴‐*O*‐acetyl‐β‐D‐allopyranosyl‐(1→2)‐β‐D‐glucopyranoside] (**38**)	−9.1	V465, V418, V420, V561, V369, Q563, and G367
4′‐*O*‐Methylhypolaetin 7‐*O*‐[6‴‐*O*‐acetyl‐β‐D‐allopyranosyl‐(1→2)‐β‐D‐glucopyranoside] (**28**)	−9.7	ASN‐469, ARG‐470, VAL‐467, VAL‐606, VAL‐608, GLY‐367, and VAL‐369

**FIGURE 3:**
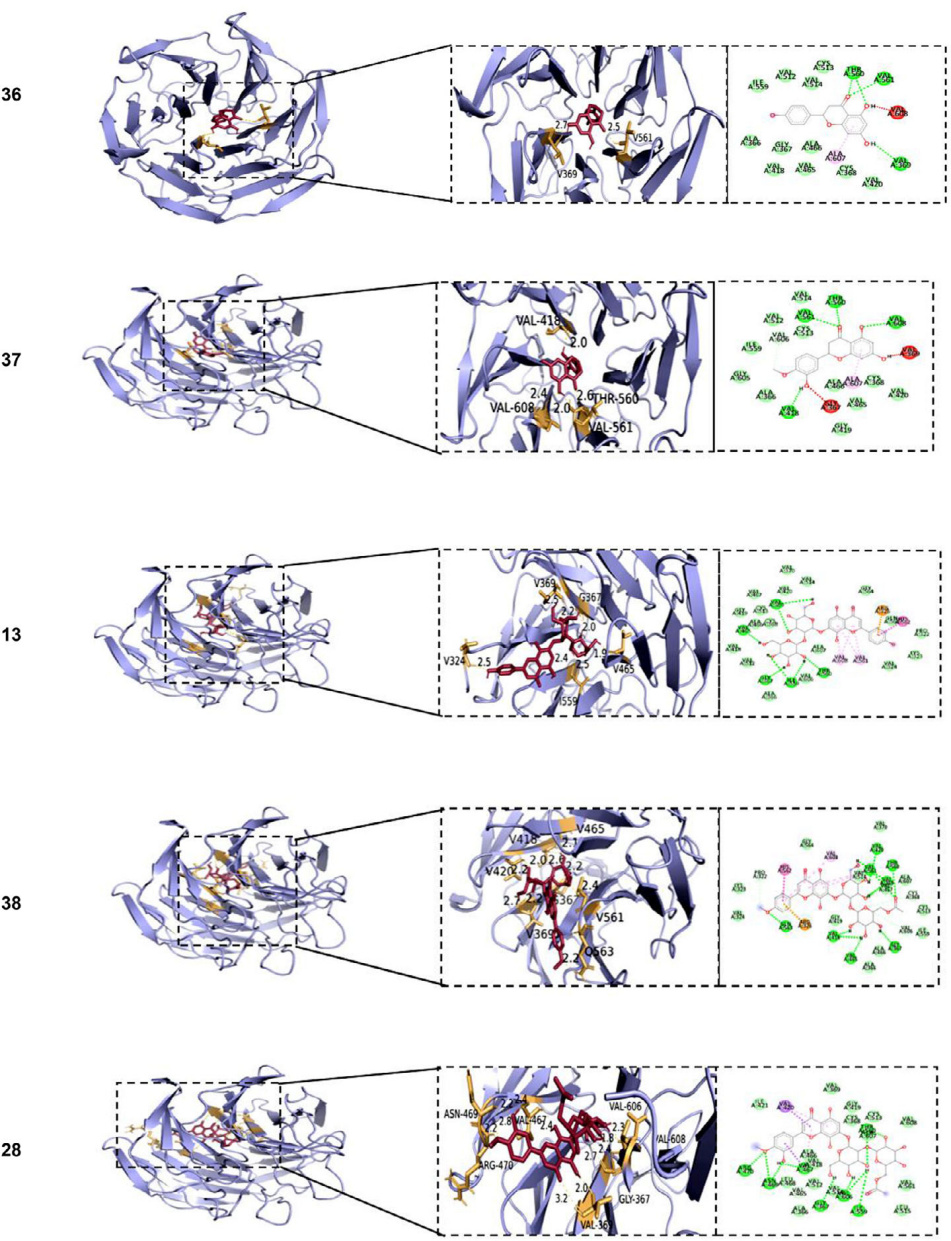
Three‐dimensional (3D) and two‐dimensional (2D) interaction diagrams of compounds **13, 28**, and **36–38** with key amino acid residues in the active site of Keap1 Kelch domain. In the 3D diagrams, the protein surface is shown in transparent gray to delineate the binding pocket boundary; compounds are depicted as yellow sticks; key interacting residues are shown as green sticks; hydrogen bonds are shown as yellow dashed lines with distances labeled in Å. In the 2D diagrams, hydrogen bonds are shown as purple dashed lines; hydrophobic interactions are represented by red arcs around the ligand atoms; polar interactions are shown as green dashed lines. Key amino acid residues and binding energies are summarized in Table 4.

The docking results revealed significant differences in the binding affinity of each compound to the target proteins. Among them, 4′‐*O*‐methylhypolaetin 7‐*O*‐[6‴‐*O*‐acetyl‐β‐D‐allopyranosyl‐(1→2)‐β‐D‐glucopyranoside] (**28**) exhibited the strongest binding capacity (binding energy: −9.7 kcal/mol), followed by isoscutellarein 7‐*O*‐β‐D‐allopyranosyl‐(1→2)‐β‐D‐glucopyranoside (**13**) (binding energy: −9.5 kcal/mol). Amino acid interaction analysis indicated that valine (VAL) family residues (e.g., VAL‐369, VAL‐418, VAL‐561, and VAL‐608) were the core binding sites for most compounds, maintaining the stability of the complex through the formation of stable hydrophobic interactions [32]. Additionally, amino acids, such as glycine (GLY‐367), asparagine (ASN‐469), and arginine (ARG‐470), participated in the formation of hydrogen bonds or polar interactions, further enhancing the binding stability between ligands and target proteins. These results are consistent with the in vitro biological activity evaluation data, confirming the reliability of the docking model [33].

### 2.5 ADMET Prediction

The ADMET properties and toxicological profiles of the compounds were predicted using SwissADME and related in silico tools (summarized in Table 5).

**TABLE 5:** Predicted ADMET properties of the compounds **13, 28**, and **36–38**.

Compounds	GI absorption	BBB permeability	P‐gp substrate	CYP inhibition	PAINS alerts	Brenk alerts	Druggability (Lipinski rule violations)
**36**	High	No	Yes	CYP1A2, CYP3A4	0	0	Yes (0)
**37**	High	No	Yes	CYP1A2	0	0	Yes (0)
**13**	Low	No	Yes	None	0	1 (Hydroquinone structure)	No (3)
**38**	Low	No	Yes	None	0	1 (Hydroquinone structure)	No (3)
**28**	Low	No	Yes	None	0	1 (Hydroquinone structure)	No (3)

#### 2.5.1 Absorption

Naringenin (**36**) and hesperetin (**37**) showed high oral gastrointestinal absorption (>80%) and moderate water solubility (naringenin: 8.74 × 10^−^^2^ mg/mL; hesperetin: 7.19 × 10^−^^2^ mg/mL), providing a good foundation for oral administration. Glycoside derivatives **13, 28**, and **38** exhibited low oral absorption efficiency owing to their large molecular weights (610.52–682.58 g/mol) and high polarity. Moreover, different prediction models showed inconsistent water solubility results for these glycosylated derivatives (isoscutellarein glycoside: 1.08–302 mg/mL). All compounds had poor skin permeability (log Kp < −6.17 cm/s), indicating that they are not suitable for topical administration [34].

#### 2.5.2 Distribution

All the tested compounds were unable to penetrate the blood–brain barrier (BBB permeability: no), suggesting that their biological effects are mainly concentrated in peripheral tissues. All the compounds were identified as P‐glycoprotein (P‐gp) substrates [34], which may lead to their efflux in intestinal epithelial cells and thereby affect in vivo bioavailability.

#### 2.5.3 Metabolism

Naringenin (**36**) inhibited two key metabolic enzymes, CYP1A2 and CYP3A4; therefore, caution should be exercised when used in combination with drugs metabolized by these enzymes to avoid potential drug–drug interaction risks. Hesperetin (**37**) inhibited CYP1A2 alone [35], resulting in a relatively lower risk of drug interactions. The three glycoside derivatives showed no inhibitory activity against major cytochrome P450 enzymes (CYP1A2, CYP2C19, CYP2C9, CYP2D6, and CYP3A4), indicating higher metabolic safety.

#### 2.5.4 Toxicity

No PAINS or Brenk toxic structural alerts were detected for naringenin (**36**) and hesperetin (**37**) [36], demonstrating good toxicological safety. All three glycoside compounds triggered 1 Brenk alert due to the presence of a hydroquinone structure [37], indicating potential toxicological risks; however, no serious toxic characteristics, such as AMES toxicity or hepatotoxicity, were found. The oral acute toxicity (LD_50_) of all compounds in rats was within the safe range, suggesting low acute toxicity.

#### 2.5.5 Druggability

Naringenin (**36**) and hesperetin (**37**) complied with Lipinski's Rule of Five (0 violations) [38] and met other in silico druggability evaluation criteria (Ghose, Veber, Egan, and Muegge rules), with a bioavailability score of 0.55, suggesting favorable physicochemical properties for oral drug development. However, it should be emphasized that these computational predictions serve as an initial guideline and do not guarantee in vivo druggability; experimental validation of pharmacokinetic properties remains essential [39]. Glycosylated derivatives violated multiple druggability rules (3 violations) because of factors such as molecular weight exceeding 500 Da, an excessive number of hydrogen bond donors/acceptors, and a topological polar surface area (TPSA) > 140 Å^2^, leading to low predicted bioavailability (bioavailability score = 0.17) and high synthetic complexity (synthetic accessibility score > 6.46).

An apparent discrepancy was observed between the favorable docking scores of the three glycosylated derivatives (**13, 28**, and **38**) with binding energies ranging from −9.1 to −9.7 kcal/mol (Table 4) and their violation of multiple Lipinski's Rule of Five criteria (Table 5). This phenomenon can be explained by several factors. First, the glycoside moieties contribute substantial molecular weight (>500 Da) and numerous hydrogen bond donors/acceptors, which are penalized by the Lipinski rules. However, Lipinski himself noted that natural products, particularly glycosides, often fall outside the Rule of Five due to the involvement of active transport mechanisms that facilitate absorption [40]. Second, the strong binding affinities observed in docking simulations reflect the ability of these glycoside derivatives to establish extensive hydrogen bonding and hydrophobic interactions with the Keap1 Kelch domain pocket, which is a measure of target affinity rather than oral bioavailability [32]. Therefore, the discrepancy between docking scores and Lipinski compliance underscores a fundamental distinction: molecular docking predicts target‐binding potential, whereas the Rule of Five predicts oral absorption likelihood. Compounds with favorable target affinity but poor predicted oral bioavailability may still be valuable as lead compounds for further optimization, or as candidates for non‐oral routes of administration.

Molecular docking is a valuable computational tool for predicting protein–ligand interactions and generating testable hypotheses. However, it is important to recognize that docking scores and predicted binding modes are approximations based on scoring functions and force fields, and do not substitute for experimental validation [31]. The binding affinities and interaction patterns reported in this study should be considered as hypotheses to be tested in future experimental studies, including surface plasmon resonance (SPR), isothermal titration calorimetry (ITC), or cellular functional assays. Additionally, although no redocking validation was performed in the present study, the use of a high‐resolution (1.80 Å) and well‐validated crystal structure (PDB: 4IFJ) [28] provides a solid structural basis for the docking analysis.

### 2.6 Verification of Binding Capacity Through Molecular Dynamics Simulation (MDS)

As shown in Figure 4A, the root–mean–square deviation (RMSD) of the KEAP1–compounds system fluctuated below 0.87 nm; this small deviation indicated high stability when the small molecules binded to the KEAP1 target protein. The radius of gyration (Rg) of the KEAP1–compounds remained relatively stable during dynamic motion, indicating that the small‐molecule–target protein complex did not undergo significant expansion or contraction during this process (Figure 1B). Upon binding of the KEAP1 compound receptors to its ligand, the solvent‐accessible surface area (SASA) of the resulting complex did not change significantly, further indicating that the protein–small‐molecules interaction could achieve a relatively stable binding. As shown in Figure 1D, the root–mean–square–fuctuation (RMSF) values for the KEAP1–compounds complex were relatively low (most values were below 0.25 nm), indicating low flexibility and high stability. The number of hydrogen bonds between small molecules and target proteins during the kinetic process was shown in Figure 1E; for KEAP1‐compounds, approximately 4–5 hydrogen bonds existed between the small molecules and the target protein, indicating strong hydrogen‐bonding interactions between the ligand and the target protein. The free energy landscape (FEL) is shown in Figure 1F; the KEAP1–compounds complexes exhibited lower free energy when PC1 (representing RMSD) was less than 0.105 and the PC2 value (representing Rg) was less than 1.715, indicating that this regional conformation represented the dominant stable conformation during the simulation. In conclusion, the KEAP1‐13, KEAP1‐28, KEAP1‐36, KEAP1‐37, and KEAP1‐38 complex systems exhibited stable binding, and these complexes possessed strong hydrogen bonding interactions. As a result, the small molecules demonstrated efficient binding to their target protein.

**FIGURE 4:**
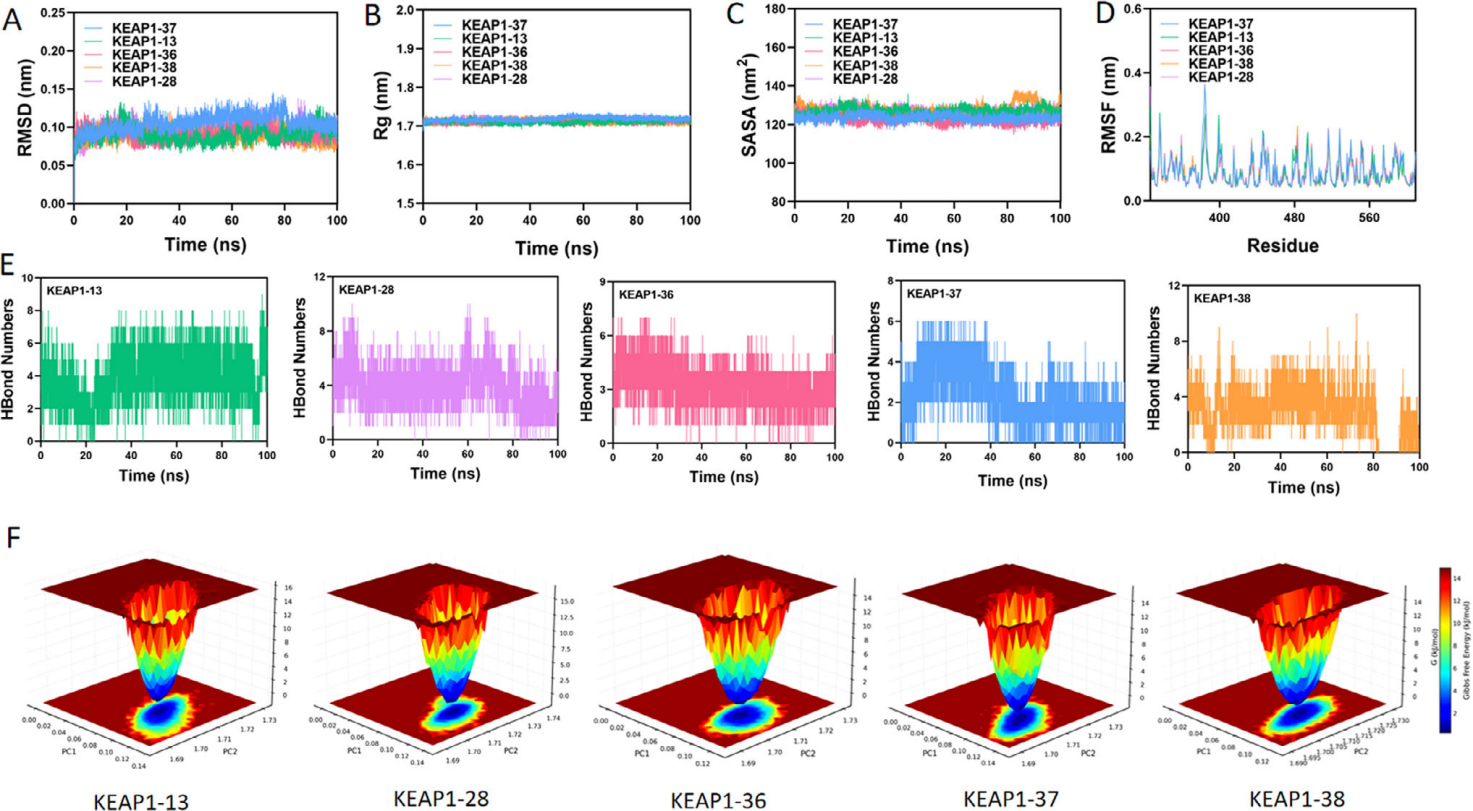
The result of MDS. (A) RMSD value of the protein–ligand complex over time. (B) Rg value of the protein–ligand complex over time. (C) SASA value of the protein–ligand complex over time. (D) RMSF value of the protein–ligand complex. (E) H Bonds value of the protein–ligand complex over time. (F) Free energy landscape plot.

## 3 Conclusions

The present study demonstrated that *S. hissarica* extracts possess considerable in vitro antioxidant activity, with the aqueous methanolic extract showing the strongest radical‐scavenging and reducing capacities in the DPPH, FRAP, and ABTS assays. The observed activities were comparable to those of the reference antioxidant ascorbic acid under the conditions tested. LC‐MS/MS analysis revealed a diverse profile of secondary metabolites, including hydroxycinnamic acid derivatives, flavonoid glycosides, and methoxylated flavones, while several flavonoid constituents were further isolated and structurally characterized using spectroscopic methods. The antioxidant activity observed in the present study may be associated, at least in part, with the presence of these phenolic constituents; however, their individual contributions and possible interactions require further investigation. The detection and characterization of previously unreported metabolites also expand the current knowledge of the phytochemical composition of *S. hissarica*. Overall, these findings provide new information on the antioxidant properties and secondary metabolites of *S. hissarica* and support further phytochemical and pharmacological investigations of its isolated constituents. Molecular docking analysis further revealed that glycosylated derivatives **13** and **28** exhibited the strongest binding affinities to the Keap1 Kelch domain, with binding energies of −9.5 and −9.7 kcal/mol, respectively. Molecular dynamics simulations also demonstrated that the binding between KEAP1 and the compounds were stable, and the resulting complexes exhibited strong hydrogen bonding interactions, indicating efficient binding of the molecules to the target protein. Experimental validation through biochemical and pharmacological assays remains essential to confirm the actual binding and functional effects of these compounds.

## 4 Experimental Section

### 4.1 Chemicals

All solvents, including LC‐MS‐grade methanol, water, acetonitrile, and formic acid, used for HPLC‐PDA/ESI‐MS analyses were purchased from Merck KGaA (Darmstadt, Germany). The following chemicals were purchased from Sigma‐Aldrich (Darmstadt, Germany): 2,2′‐azino‐bis (3‐ethylbenzothiazoline‐6‐sulfonic acid) (ABTS), 1,1‐diphenyl‐2‐picrylhydrazyl (DPPH), ascorbic acid, and trolox. All chemicals were of analytical grade.

### 4.2 Plant Materials

Aerial parts of *S. hissarica* Regel (N2016023) were collected from the Chimgan mountains (41°31'52.0“N 70°01'54.0”E) (Tashkent region, Uzbekistan) (Figure S30). The plant was collected at the fully grown stage during spring to ensure maximum yield. The plant was authenticated by Dr. Olim Nigmatullaev, and voucher samples were deposited at the Herbarium of the Institute of Chemistry of Plant Substances, Academy of Sciences of Uzbekistan.

### 4.3 HPLC‐QqQ‐MS/MS Analysis

HPLC‐PDA/ESI‐MS analysis was carried out on a Shimadzu HPLC system (Shimadzu, Duisburg, Germany) equipped with a CBM‐20A controller, two LC‐30AD dual‐plunger parallel‐flow pumps, a CTO‐20A column oven, a SIL‐30AC autosampler, and an SPD‐M30A photodiode array (PDA) detector coupled to an LCMS‐8050 mass spectrometer equipped with an electrospray ionization (ESI) source (Shimadzu, Japan) [41]. The separation of phenolic compounds achieved using an Ascentis Express C18 column (150 mm × 2.1 mm I.D., 2.7 µm particle size; Merck KGaA, Darmstadt, Germany). The mobile phase consisted of water containing 0.1% formic acid (pH 3.0; solvent A) and acetonitrile containing 0.1% formic acid (solvent B). The gradient elution program was as follows: 0–10 min: 0%–10%B; 10–20 min: 10%–11%B; 20–30 min: 11%–15% B; 30–50 min: 15%–18%B; 50–65 min: 18%–23%B; and 65–70 min: 23%–100%B. The flow rate was maintained at 0.5 mL/min, with an injection volume of 2 µL, and an oven temperature of 30°C. PDA detection was conducted over the wavelength range 200–400 nm, using a sampling frequency of 12.5 Hz and a time constant of 0.16 s. Mass spectrometric measurements were performed both in both positive (+) and negative (‐) ESI modes. The interface, desolvation, and heat block temperatures were set at 350°C, 300°C, and 300°C, respectively. Nitrogen was used as both the nebulizing and drying gas at flow rates of 1.5 and 15 L/min, respectively. Data were collected over an *m/z* range of 100–1000 in both ionization modes. Instrument control, data acquisition, and processing were performed using LabSolutions software, version 5.97 (Shimadzu, Duisburg, Germany).

### 4.4 NMR Analysis

NMR measurements were performed at 25°C on a JEOL JNM‐ECZ600R 600 MHz NMR spectrometer and a Bruker Avance III HD 400 MHz NMR spectrometer [42, 43]. The 400 MHz spectrometer was equipped with a nitrogen‐cooled Prodigy cryoplatform. The ^1^H and ^13^C NMR frequencies of tetramethylsilane (TMS; *δ*_H_ = *δ*_C_ = 0.000 ppm) served as the reference frequencies for the calculation of the ^1^H and ^13^C NMR chemical shift scales. Pyridine‐*d*_5_ and DMSO‐*d*_6_ were used as solvents for the preparation of NMR samples. For the samples dissolved in DMSO‐*d*_6_, the ^1^H and ^13^C chemical shift axes were additionally shifted using, respectively, the residual signal of the non‐perdeuterated solvent signal (DMSO‐*d*_5_; *δ*_H_ = 2.500 ppm) and the perdeuterated solvent signal (DMSO‐*d*_6_; *δ*_C_ = 39.52 ppm) being secondary standard signals. In the case of pyridine‐*d*_5_ solutions, TMS was used as an internal standard, and the spectra were shifted accordingly (TMS: *δ*_H_ = *δ*_C_ = 0.000 ppm). Standard ^1^H, ^13^C, ^1^H–^1^H correlation spectroscopy (COSY), ^1^H–^13^C heteronuclear single‐quantum coherence (HSQC), and ^1^H–^13^C heteronuclear multiple bond correlation (HMBC) experiments were performed with acquisition parameters set as appropriate for each sample (the acquisition and processing parameters are available from the corresponding author upon reasonable request).

### 4.5 Extraction and Isolation

Air‐dried and powdered aerial parts (890 g) of *S. hissarica* were extracted with methanol‐water (7:1, v/v) (4 × 5 L) at room temperature for 96 h. The combined extracts were filtered and concentrated under reduced pressure at 40°C to obtain an aqueous methanolic extract (99.7 g, 11.2%, w/w). A portion of the extract (95 g) was suspended in distilled water (1 L) and successively partitioned with benzene (3 × 1.5 L), ethyl acetate (3 × 1 L), and *n*‐butanol (3 × 0.7 L). Evaporation under reduced pressure afforded benzene (5.3 g), ethyl acetate (36.2 g), and *n*‐butanol (46.8 g) fractions. Based on TLC analysis, the ethyl acetate and *n*‐butanol fractions were selected for further chromatographic separation.

The ethyl acetate fraction (35 g) was subjected to column chromatography over silica gel (column size: 6 × 80 cm) Elution was performed using a stepwise gradient of chloroform–methanol (CHCl_3_–MeOH), starting from 100:0 to 0:100 (v/v). Fractions of 250 mL were collected and monitored by TLC on silica gel 60 F_254_ plates using CHCl_3_–MeOH–H_2_O (70:30:5, v/v/v) as the developing mobile system. Spots were visualized under UV light (254 and 365 nm) and by spraying with anisaldehyde. Fractions were pooled based on similar TLC profiles to yield eight combined fractions (E1–E8). Subfraction E3 was further purified by silica gel column chromatography using chloroform–methanol (95:5, v/v) to afford naringenin (**36**) (18 mg). Subfraction E4 was repeatedly chromatographed over silica gel using chloroform–methanol (90:10, v/v), followed by recrystallization from methanol to yield hesperetin (**37**) (14 mg).

The *n*‐butanol fraction (45 g) was chromatographed on a Diaion HP‐20 column (15 × 60 cm). Elution was carried out using a stepwise gradient of H_2_O‐MeOH (0%, 25%, 50%, 75%, and 100% MeOH) to obtain five fractions (B1–B5). Fraction B5 further separated by silica gel column chromatography using chloroform–methanol–water (65:35:10, v/v/v). Repeated purification afforded isoscutellarein 7‐*O*‐β‐D‐allopyranosyl‐(1→2)‐β‐D‐glucopyranoside (**13**) (22 mg). Fraction B4 was subjected to silica gel column chromatography using EtOAc–MeOH–H_2_O (100:13:10, v/v/v). Repeated purification yielded 4′‐*O*‐methylisoscutellarein 7‐*O*‐[6‴‐*O*‐acetyl‐β‐D‐allopyranosyl‐(1→2)‐β‐D‐glucopyranoside] (**38**) (4 mg). Fraction B3 was chromatographed over silica gel using CHCl_3_–MeOH–H_2_O (70:30:5, v/v/v) to afford 3′‐hydroxy‐4′‐*O*‐methylisoscutellarein 7‐*O*‐[6‴‐*O*‐acetyl‐β‐D‐allopyranosyl‐(1→2)‐β‐D‐glucopyranoside] (**28**) (1.7 mg). The structures of the isolated compounds were elucidated by spectroscopic methods (including 1D and 2D NMR) and HR‐ESI‐MS, and by comparison with literature data [19, 23].

*Naringenin* (**36**), C_15_H_12_O_5_, *M* = 272.253. HR‐MS: *m/z* 273.07 [M+H]^+^ (calc. 273.0763). ^1^H NMR (600.2 MHz, DMSO‐*d*_6_, *δ*/ppm, *J*/Hz) and ^13^C NMR (150.9 MHz, DMSO‐*d*_6_, *δ*/ppm) data are presented in Table S1 and Figures S2–S5.

*Hesperetin* (**37**), (3′,5,7‐trihydroxy‐4′‐methoxyflavan‐4‐one), C_16_H_14_O_6_, *M* = 302.28. HR‐MS: *m/z* 303.0901 [M+H]^+^ (calc. 303.0869). ^1^H NMR (600.2 MHz, DMSO‐*d*_6_, *δ*/ppm, *J*/Hz) and ^13^C NMR (150.9 MHz, DMSO‐*d*_6_, *δ*/ppm) data are presented in Table S2 and Figures S6–S9.

*Isoscutellarein 7‐O‐β‐D‐allopyranosyl‐(1→2)‐glucopyranoside* (**13**), C_27_H_30_O_16_, *M* = 610.50. HR‐MS: *m/z* 611.1627 [M+H]^+^ (calc. 611.1612). ^1^H NMR (600.2 MHz, pyridine‐*d*_5_, *δ*/ppm, *J*/Hz) and ^13^C NMR (150.9 MHz, pyridine‐*d*_5_, *δ*/ppm) data are presented in Table S3 and Figures S10–S14.

*4′‐O‐Methylisoscutellarein 7‐O‐[6‴‐O‐acetyl‐β‐D‐allopyranosyl‐(1→2)‐β‐D‐glucopyranoside* (**38**), C_30_H_34_O_17_, *M* = 666.58. HR‐MS: *m/z* 667.1941 [M+H]^+^ (calc. 667.1874). ^1^H NMR (600.2 MHz, pyridine‐*d*_5_, *δ*/ppm, *J*/Hz) and ^13^C NMR (150.9 MHz, pyridine‐*d*_5_, *δ*/ppm) data are presented in Table S4 and Figures S15–S24.

*4′‐O‐Methylhypolaetin 7‐O‐[6‴‐O‐acetyl‐β‐D‐allopyranosyl‐(1→2)‐β‐D‐glucopyranoside] (syn. 3′‐hydroxy‐4′‐O‐methylisoscutellarein 7‐O‐[6‴‐O‐acetyl‐β‐D‐allopyranosyl‐(1→2)‐β‐D‐glucopyranoside]) (* ***28***), C_30_H_34_O_18_, *M* = 682.58. HR‐MS: m/z 683.1801 [M+H]^+^ (calc. 683.1823). ^1^H NMR (600.2 MHz, pyridine‐d_5_, *δ*/ppm, J/Hz) and ^13^C NMR (150.9 MHz, pyridine‐d_5_, *δ*/ppm) data are presented in Table S5 and Figures S25–S29.

### 4.6 In Vitro Antioxidant Activity

#### 4.6.1 Free Radical Scavenging Activity (DPPH)

The free radical scavenging activity of different *S. hissarica* extracts was evaluated using the 1,1‐diphenyl‐2‐picryl hydrazyl (DPPH) with minor modifications [44]. A 0.1 mM DPPH solution was freshly prepared by dissolving 4 mg of DPPH in 100 mL of methanol and was kept at 4°C in the dark until use. For each extract, a stock solution at 500 µg/mL was prepared by dissolving 5 mg of extract in 10 mL of methanol. Serial dilutions were then performed to obtain final concentrations of 12, 25, 50, 100, 250, and 500 µg/mL. For each concentration, 2 mL of the extract solution was transferred into an optical glass cuvette, followed by the addition 3 mL of the 0.1 mM DPPH solution. After 30 min of incubation in the dark, the absorbance was recorded at 517 nm using a UV–visible (UV) spectrophotometer. Ascorbic acid was employed as the reference compound. A stock solution of ascorbic acid (1000 µg/mL) was prepared by dissolving 2 mg in 2 mL of distilled water, followed by serial dilution to obtain concentrations of 12, 25, 50, 100, 250, and 500 µg/mL. Appropriate solvent blanks were used for each extract (MeOH, CHCl_3_, EtOAc, MeOH:H_2_O (7:3, v/v), and BuOH). A mixture of 3 mL of 0.1 mM DPPH and 100 µL of the respective solvent (100 µL MeOH for methanol extract, 100 µL CHCl_3_ for chloroform extract, 100 µL EtOAc for ethyl acetate, 100 µL MeOH:H_2_O (7:3, v/v) for aqueous methanolic extract, and 100 µL BuOH for butanol extract) was used as the control. All measurements were conducted in triplicate. The degree of inhibition (expressed as a percentage) was plotted against concentration, and IC_50_ values were obtained from the resulting plots.

$$\mathrm{DPPH}\;\mathrm{inhibition}\left(\%\right)=[\left(A_{\mathrm{control}}-A_{\mathrm{sample}}\right)/A_{\mathrm{control}}\times 100$$

where, *A*_control_ is the absorbance of the mixture of methanol/ethanol/water and DPPH solution, whereas *A*_sample_ is the absorbance of the sample extract and DPPH solution.

The percentage inhibition was plotted against the corresponding extract concentration, and IC_50_ values were calculated from the resulting dose–response curves.

#### 4.6.2 ABTS (2,2′‐Azino‐bis (3‐ethylbenzothiazloine‐6‐sulfonic acid)) Radical Cation Scavenging Activity

The total antioxidant activity of the tested samples was evaluated using the ABTS assay according to the procedure reported by Banothu et al. with minor modifications [45]. The ABTS solution was prepared by mixing 7 mmol of ABTS and 2.45 mmol of potassium persulfate (K_2_S_2_O_8_) in water (in a 1:1 ratio), followed by incubation at room temperature in the dark for 12–16 h. The radical cation formed was further diluted with ethanol (1:1, v/v) to obtain an absorbance of 0.700 at 734 nm, as measured using a UV–Vis spectrophotometer. For the assay, 2 mL of the ABTS solution was mixed with 100 µL of each test sample at different concentrations. The samples were mixed thoroughly and incubated at room temperature for 10 min, after which the absorbance was measured at 734 nm. The reactivity of various concentrations of each solvent extract was compared with that of ascorbic acid. Measurements were performed in triplicate, and IC_50_ values were calculated from the corresponding concentration–response curves. The percentage of scavenging of ABTS^•+^ radical was determined for extract and standard concentrations ranging from 50 to 300 µg/mL and expressed as percentage inhibition according to the following equation:

$$\begin{array}{ccc} & & ABTS\;radical\;scavenging\;activity\;\left(\%\right)\\ & & \;=[\left(OD_{\mathrm{control}}-OD_{\mathrm{sample}}\right)/OD_{\mathrm{control}}\times 100 \end{array}$$

where OD_control_ represents the absorbance of the ABTS•+ solution without the test sample and OD_sample_ represents the absorbance measured in the presence of the tested extract or standard.

#### 4.6.3 Ferric Reducing Antioxidant Power (FRAP) Method

The FRAP assay was carried out following the procedure reported by Gad et al. [12]. Briefly, 100 µL of each sample solution was mixed 2 mL of freshly prepared FRAP reagent, consisting of 0.3 M acetate buffer (pH 3.6), 10 mM 2,4,6‐tris(2‐pyridyl)‐*s*‐triazine (TPTZ) prepared in 40 mM HCl, and 20 mM ferric chloride (FeCl_3_), combined in a 10:1:1 (v/v/v) ratio. The reaction mixtures were incubated at room temperature for 30 min, after which the absorbance was measured at 593 nm. This absorbance was used to estimate the reducing power of the samples based on their ability to convert Fe^3^^+^ to Fe^2^^+^, resulting in the formation of the Fe^2^^+^–TPTZ complex. Trolox was used as the reference standard, and the IC_50_ values were calculated to enable comparison of the antioxidant activity of the tested samples.

#### 4.6.4 Statistical Analysis

All experiments were performed in triplicate, and the results are expressed as mean ± standard deviation (SD). Statistical analyses were performed using one‐way analysis of variance (ANOVA), followed by Tukey's post hoc multiple‐comparison test. A *p*‐value <0.05 was considered statistically significant. All statistical analyses were performed using GraphPad Prism version 8.0.1 (GraphPad Software, Inc., San Diego, CA, USA).

### 4.7 In Silico Modelling and Molecular Dynamics Studies

#### 4.7.1 In Silico Molecular Modelling Studies

Molecular docking experiments were performed using AutoDockTools 1.5.6 software to evaluate the binding affinity between target compounds and proteins. The binding free energy data from molecular docking simulations are presented directly as calculated binding energy values. The ADMET properties and toxicological profiles of the compounds were predicted using SwissADME [34] and related in silico tools. The predicted ADMET pharmacokinetic properties are represented as composite scores derived from multiple‐algorithm models, which were then subjected to descriptive statistical analysis.

#### 4.7.2 Verification of the Binding Model Using MDS

The processing of receptor protein and ligand structures, as well as protein–ligand complex molecular dynamics simulation analysis methods were consulted to our previous papers [46]. Briefly, during the construction of the MDS system, the TIP3P water model was employed for solvation treatment; energy minimization was then performed sequentially using the steepest descent method and the conjugate gradient method. Once the system had reached full equilibrium, MDS without positional constraints were conducted. Upon completion of the simulations, subsequent data processing was carried out using the integrated analysis tools provided by GROMACS 2022.2.

## Author Contributions

**Elbek A. Mukhammadiev**: methodology, formal analysis, investigation, and writing – original draft. **Michal Šoral**: methodology, investigation, software, data curation, and writing – review and editing. **Francesco Cacciola**: investigation, software, data curation, and writing – review and editing. **Katia Arena**: methodology, software, and resources. **Chao Liu**: methodology, software, and resources. **Mengqi Zhang**: investigation, software, data curation, and resources. **Ruirui Zhang**: methodology, software, and resources. **Doniyor Siddikov**: investigation, software, data curation, and resources. **Sobirdjan A. Sasmakov**: investigation, software, data curation, and resources. **Nilufar Z. Mamadalieva**: conceptualization, supervision, project administration, resources, writing – original draft, and writing – review, and editing. All authors have read and agreed to the published version of the manuscript.

## Conflicts of Interest

The authors declare no conflicts of interest.

## Supporting information




**Supporting File 1**: cbdv71813‐sup‐0001‐SuppMat.docx.

## Data Availability

The data that support the findings of this study are available in the Supporting Information of this article.
