# Supplementary material for: Phytochemical Investigation of Stachys hissarica: Antioxidant Activity, Flavonoid Characterization, and In Silico Analysis
Source: Chem Biodivers. 2026 Oct 15;23(10):e71813. doi: 10.1002/cbdv.71813 (PMC13670987; doi:10.1002/cbdv.71813)
Supplement: Supplementary file 1 [file CBDV-23-e71813-s001.docx]

**Supporting Information**

Phytochemical Investigation of *Stachys hissarica*: Antioxidant Activity, Flavonoid Characterization, and *In Silico* Analysis

Elbek A. Mukhammadiev^a^, Michal Šoral^b^, Francesco Cacciola^c^, Katia Arena^c^, Chao Liu^d^, Mengqi Zhang^d^, Ruirui Zhang^d^, Doniyor R. Siddikov^a^, Sobirdjan A. Sasmakov^a^, Nilufar Z. Mamadalieva^a,e*^

^a^ Institute of the Chemistry of Plant Substances, Uzbekistan Academy of Sciences, Mirzo Ulugbek Str. 77, 100170 Tashkent, Uzbekistan; [elbekmuhammadiyev93@gmail.com](mailto:elbekmuhammadiyev93@gmail.com) (0009-0004-1765-4833), [drsiddiqov@mail.ru](mailto:drsiddiqov@mail.ru) (0000-0002-0548-2665), [sasmakov@web.de](mailto:sasmakov@web.de) (0000-0003-1863-7117), [nmamadalieva@yahoo.com](mailto:nmamadalieva@yahoo.com) (0000-0003-1756-3638)

^b^ Analytical Department, Institute of Chemistry, Slovak Academy of Sciences, Dúbravská cesta 9, SK-845 38 Bratislava, Slovak Republic; [michal.soral@savba.sk](mailto:michal.soral@savba.sk) (0000-0002-4537-7173)

^c^ Messina Institute of Technology, c/o Department of Chemical, Biological, Pharmaceutical and Environmental Sciences, Former Veterinary School, University of Messina, 98168 Messina, Italy; [cacciolaf@unime.it](mailto:cacciolaf@unime.it) (0000-0003-1296-7633), [katia.arena@unime.it](mailto:katia.arena@unime.it) (0000-0002-6441-1461)

^d^ Key Laboratory of Novel Food Resources Processing, Ministry of Agriculture and Rural Affairs/Institute of Food & Nutrition Science and Technology, Shandong Academy of Agricultural Sciences/Tea Research Institute, Shandong Academy of Agricultural Sciences. Jinan 250100, China; [liuchao555@126.com](mailto:liuchao555@126.com) (0000-0003-2680-1810), mengqi139@126.com (0000-0002-1432-3471), [zhangrui15868@163.com](mailto:zhangrui15868@163.com) (0000-0002-3328-1318)

^e^ Faculty of Medicine, Alfraganus University, Yuqori Qoraqamish Str. 2a, 100190 Tashkent, Uzbekistan; [n.mamadaliyeva@afu.uz](mailto:n.mamadaliyeva@afu.uz) (0000-0003-1756-3638)

*Correspondence: [n.mamadaliyeva@afu.uz](mailto:n.mamadaliyeva@afu.uz), nmamadalieva@yahoo.com

**Table S1-S5.** NMR characterization of the isolated compounds **13, 28,** and **36-38**

**Figure S1.** Polyphenolic profile of methanolic-aqueous extract of *S. hissarica* by HPLC-PDA/MS

**Figure S2-S29.** 1D and 2D spectra of the isolated compounds **13, 28,** and **36-38**

**Figure S30.** *Stachys hissarica* Regel (N2016023) were collected from the Chimgan mountains (41°31'52.0"N 70°01'54.0"E) (Tashkent region, Uzbekistan)

**Table S1.** NMR data and signal assignment of naringenin (**36**) (DMSO-*d*_6_, 600.2 MHz for ^1^H, 150.9 MHz for ^13^C)

|  | | | |
| --- | --- | --- | --- |
| Position | ^1^H chemical shift *δ*_H_ / ppm | Signal multiplicity; *J*_HH_ / Hz | ^13^C chemical shift *δ*_C_ / ppm |
| 1 | - | - | - |
| 2 | 5.310 | dd; 13.0, 2.9 | 78.37 |
| 3 | 2.648 | dd; 17.0, 2.9 | 42.41 |
|  | 3.063 | dd; 17.0, 13.0 |  |
| 4 | - | - | 195.17 |
| 5 | - | - | 163.64 |
| 6 | 5.812 | d; 2.1 | 95.83 |
| 7 | - | - | 166.61 |
| 8 | 5.821 | d; 2.1 | 94.81 |
| 9 | - | - | 162.63 |
| 10 | - | - | 101.62 |
| 1’ | - | - | 128.49 |
| 2’ | 7.228 | m | 127.51 |
| 3’ | 6.754 | m | 115.04 |
| 4’ | - | - | 157.69 |
| 5’ | 6.754 | m | 115.04 |
| 6’ | 7.228 | m | 127.51 |
| 5-OH | 12.002 | s | - |
| 7-OH | 10.448 | br s | - |
| 4’-OH | 9.296 | br s | - |

**Table S2.** NMR data and signal assignment of hesperetin (**37**) (DMSO-*d*_6_, 600.2 MHz for ^1^H, 150.9 MHz for ^13^C)

|  | | | |
| --- | --- | --- | --- |
| Position | ^1^H chemical shift *δ*_H_ / ppm | Signal multiplicity; *J*_HH_ / Hz | ^13^C chemical shift *δ*_C_ / ppm |
| 1 | - | - | - |
| 2 | 5.297 | dd; 13.3, 3.1 | 78.17 |
| 3 | 2.669 | dd; 17.0, 3.1 | 42.43 |
|  | 3.027 | dd; 17.0, 13.3 |  |
| 4 | - | - | 194.99 |
| 5 | - | - | 163.61 |
| 6 | 5.814 | d; 2.1 | 95.84 |
| 7 | - | - | 166.60 |
| 8 | 5.839 | d; 2.1 | 94.82 |
| 9 | - | - | 162.48 |
| 10 | - | - | 101.62 |
| 1’ | - | - | 131.08 |
| 2’ | 6.883 | d; 2.1 | 113.64 |
| 3’ | - | - | 146.66 |
| 4’ | - | - | 147.62 |
| 5’ | 6.829 | d; 8.1 | 111.60 |
| 6’ | 6.795 | dd; 8.1, 2.1 | 116.91 |
| 5-OH | 11.991 | s | - |
| 7-OH | 10.433 | br s | - |
| 3’-OH | 8.764 | br s | - |
| 4’-OMe | 3.814 | s | 55.40 |

**Table S3.** NMR data and signal assignment of isoscutellarein 7-*O*-β-D-allopyranosyl-(1→2)-β-D-glucopyranoside (**13**) (pyridine-*d*_5_, 600.2 MHz for ^1^H, 150.9 MHz for ^13^C)

|  | | | |
| --- | --- | --- | --- |
| Position | ^1^H chemical shift *δ*_H_ / ppm | Signal multiplicity; *J*_HH_ / Hz | ^13^C chemical shift *δ*_C_ / ppm |
| 1 | - | - | - |
| 2 | - | - | 164.97 |
| 3 | 6.952 | s | 103.63 |
| 4 | - | - | 183.37 |
| 5 | - | - | 154.10 |
| 6 | 7.252 | s | 100.22 |
| 7 | - | - | 152.46 |
| 8 | - | - | 128.95 |
| 9 | - | - | 145.62 |
| 10 | - | - | 106.95 |
| 1’ | - | - | 122.34 |
| 2’ | 8.125 | m | 129.20 |
| 3’ | 7.189 | m | 116.88 |
| 4’ | - | - | 162.87 |
| 5’ | 7.189 | m | 116.88 |
| 6’ | 8.125 | m | 129.20 |
| 1’’ | 5.651 | d; 7.8 | 101.99 |
| 2’’ | 4.270 | dd; 9.4, 7.8 | 83.63 |
| 3’’ | 4.414 | m | 77.68 |
| 4’’ | 4.299 | m | 70.60 |
| 5’’ | 4.061 | ddd; 9.7, 4.9, 2.3 | 78.98 |
| 6’’ | 4.391 | dd; 12.1, 4.9 | 61.89 |
|  | 4.487 | dd; 12.1, 2.3 |  |
| 1’’’ | 5.860 | d; 8.0 | 104.49 |
| 2’’’ | 4.135 | dd; 8.0, 2.9 | 73.49 |
| 3’’’ | 4.753 | t; 2.9 | 72.66 |
| 4’’’ | 4.237 | dd; 9.9, 2.9 | 68.88 |
| 5’’’ | 4.668 | ddd; 9.9, 5.6, 2.4 | 76.84 |
| 6’’’ | 4.424 | dd; 11.8, 5.6 | 62.83 |
|  | 4.624 | dd; 11.8, 2.4 |  |
| 5’-OH | 13.087 | br s | - |

**Table S4.** NMR data and signal assignment of 4'-*O*-methylisoscutellarein 7-*O*-[6'''-*O*-acetyl-β-D-allopyranosyl-(1→2)-β-D-glucopyranoside] (**38**) (pyridine-*d*_5_, 600.2 MHz for ^1^H, 150.9 MHz for ^13^C)

|  | | | |
| --- | --- | --- | --- |
| Position | ^1^H chemical shift *δ*_H_ / ppm | Signal multiplicity; *J*_HH_ / Hz | ^13^C chemical shift *δ*_C_ / ppm |
| 1 | - | - | - |
| 2 | - | - | 164.54 |
| 3 | 7.008 | s | 104.36 |
| 4 | - | - | 183.43 |
| 5 | - | - | 153.80 |
| 6 | 7.337 | s | 101.10 |
| 7 | - | - | 151.61 |
| 8 | - | - | 129.43 |
| 9 | - | - | 144.75 |
| 10 | - | - | 107.39 |
| 1’ | - | - | 124.00 |
| 2’ | 8.202 | m | 128.99 |
| 3’ | 7.139 | m | 114.97 |
| 4’ | - | - | 163.18 |
| 5’ | 7.139 | m | 114.97 |
| 6’ | 8.202 | m | 128.99 |
| 1’’ | 5.551 | d; 7.8 | 102.43 |
| 2’’ | 4.240 | dd; 9.2, 7.8 | 84.19 |
| 3’’ | 4.384 | t; 9.2 | 77.63 |
| 4’’ | 4.324 | dd; 9.5, 9.2 | 70.59 |
| 5’’ | 4.048 | ddd; 9.5, 4.9, 2.4 | 79.11 |
| 6’’ | 4.404 | dd; 12.2, 4.9 | 61.88 |
|  | 4.498 | dd; 12.2, 2.3 |  |
| 1’’’ | 5.823 | d; 8.0 | 104.78 |
| 2’’’ | 4.157 | dd; 8.1, 2.9 | 73.15 |
| 3’’’ | 4.782 | t; 2.8 | 72.41 |
| 4’’’ | 4.200 | dd; 9.9, 2.8 | 68.56 |
| 5’’’ | 4.748 | ddd; 10.0, 5.6, 2.0 | 73.56 |
| 6’’’ | 4.898 | dd; 12.0, 5.6 | 65.05 |
|  | 5.020 | dd; 12.0, 2.0 |  |
| 5-OH | 13.019 | br s | - |
| 4’-OMe | 3.731 | s | 55.52 |
| 6’’’-OAc-1 | 2.085 | s | 20.92 |
| 6’’’-OAc-2 | - | - | 171.31 |

**Table S5.** NMR data and signal assignment of 4'-*O*-methylhypolaetin 7-*O*-[6'''-*O*-acetyl-β-D-allopyranosyl(1→2)-β-D-glucopyranoside] (**28**) (pyridine-*d*_5_, 600.2 MHz for ^1^H, 150.9 MHz for ^13^C)

|  | | | |
| --- | --- | --- | --- |
| Position | ^1^H chemical shift *δ*_H_ / ppm | Signal multiplicity; *J*_HH_ / Hz | ^13^C chemical shift *δ*_C_ / ppm |
| 1 | - | - | - |
| 2 | - | - | 164.95 |
| 3 | 7.072 | s | 104.53 |
| 4 | - | - | 183.47 |
| 5 | - | - | 153.78 |
| 6 | 7.317 | s | 101.14 |
| 7 | - | - | 151.60 |
| 8 | - | - | 129.51 |
| 9 | - | - | 144.93 |
| 10 | - | - | 107.47 |
| 1’ | - | - | 124.74 |
| 2’ | 8.084 | d; 2.2 | 114.66 |
| 3’ | - | - | 148.61 |
| 4’ | - | - | 152.21 |
| 5’ | 7.106 | d; 8.6 | 112.22 |
| 6’ | 7.847 | dd; 8.6, 2.2 | 119.31 |
| 1’’ | 5.555 | d; 7.7 | 102.41 |
| 2’’ | 4.238 | dd; 9.2, 7.7 | 83.77 |
| 3’’ | 4.375 | t; 9.2 | 77.62 |
| 4’’ | 4.322 | dd; 9.4, 9.2 | 70.62 |
| 5’’ | 4.035 | ddd; 9.4, 4.9, 2.3 | 79.08 |
| 6’’ | 4.401 | dd; 12.2, 4.9 | 61.87 |
|  | 4.489 | dd; 12.2, 2.3 |  |
| 1’’’ | 5.819 | d; 8.0 | 104.53 |
| 2’’’ | 4.162 | dd; 8.0, 2.8 | 73.01 |
| 3’’’ | 4.776 | t; 2.8 | 72.45 |
| 4’’’ | 4.170 | dd; 10.0, 2.8 | 68.56 |
| 5’’’ | 4.719 | ddd; 10.0, 5.6, 2.0 | 73.56 |
| 6’’’ | 4.860 | dd; 12.0, 5.6 | 64.95 |
|  | 4.988 | dd; 12.0, 2.0 |  |
| 5-OH | 13.054 | s | - |
| 3’-OH | 11.674^a^ | br s | - |
| 4’-OMe | 3.782 | s | 55.87 |
| 4’’-OH | 7.710 | br s | - |
| 3’’’-OH | 7.391 | br s | - |
| 4’’’-OH | 6.743 | br s | - |
| 6’’’-OAc-1 | 2.130 | s | 20.93 |
| 6’’’-OAc-2 | - | - | 171.36 |

^a^ Assigned only tentatively; no correlations were observable.

**Figure S1.** Polyphenolic profile of methanolic-aqueous extract of *S. hissarica* by HPLC-PDA/MS. The numbering of peaks is consistent with the numbering of compounds in the main text and in Table 2

**Figure S2.** ^1^H NMR spectrum of naringenin (**36**) (DMSO-*d*_6_, 600 MHz)

**Figure S3.** ^13^C NMR spectrum of naringenin (**36**) (DMSO-*d*_6_, 151 MHz)

**Figure S4.** ^1^H-^13^C HSQC NMR spectrum of naringenin (**36**) (DMSO-*d*_6_, 600/151 MHz)

**Figure S5.** ^1^H-^13^C HMBC NMR spectrum of naringenin (**36**) (DMSO-*d*_6_, 600/151 MHz)

**Figure S6.** ^1^H NMR spectrum of hesperetin (**37**) (DMSO-*d*_6_, 600 MHz)

**Figure S7.** ^13^C NMR spectrum of hesperetin (**37**) (DMSO-*d*_6_, 151 MHz)

**Figure S8.** ^1^H-^13^C HSQC NMR spectrum of hesperetin (**37**) (DMSO-*d*_6_, 600/151 MHz)

**Figure S9.** ^1^H-^13^C HMBC NMR spectrum of hesperetin (**37**) (DMSO-*d*_6_, 600/151 MHz)

**Figure S10.** ^1^H NMR spectrum of isoscutellarein 7-*O*-β-D-allopyranosyl-(1→2)-β-D-glucopyranoside (**13**) (pyridine-*d*_5_, 600 MHz)

**Figure S11.** ^13^C NMR spectrum of isoscutellarein 7-*O*-β-D-allopyranosyl-(1→2)-β-D-glucopyranoside (**13**) (pyridine-*d*_5_, 151 MHz)

**Figure S12.** ^1^H-^1^H COSY NMR spectrum of isoscutellarein 7-*O*-β-D-allopyranosyl-(1→2)-β-D-glucopyranoside (**13**) (pyridine-*d*_5_, 600/600 MHz)

**Figure S13.** ^1^H-^13^C HSQC NMR spectrum of isoscutellarein 7-*O*-β-D-allopyranosyl-(1→2)-β-D-glucopyranoside (**13**) (pyridine-*d*_5_, 600/151 MHz)

**Figure S14.** ^1^H-^13^C HMBC NMR spectrum of isoscutellarein 7-*O*-β-D-allopyranosyl-(1→2)-β-D-glucopyranoside (**13**) (pyridine-*d*_5_, 600/151 MHz)

**Figure S15.** ^1^H NMR spectrum of 4'-*O*-methylisoscutellarein 7-*O*-[6'''-*O*-acetyl-β-D-allopyranosyl-(1→2)-β-D-glucopyranoside] (**38**) (pyridine-*d*_5_, 600 MHz)

**Figure S16.** ^13^C NMR spectrum of 4'-*O*-methylisoscutellarein 7-*O*-[6'''-*O*-acetyl-β-D-allopyranosyl-(1→2)-β-D-glucopyranoside] (**38**) (pyridine-*d*_5_, 151 MHz)

**Figure S17.** ^1^H-^1^H COSY NMR spectrum of 4'-*O*-methylisoscutellarein 7-*O*-[6'''-*O*-acetyl-β-D-allopyranosyl-(1→2)-β-D-glucopyranoside] (**38**) (pyridine-*d*_5_, 600/600 MHz)

**Figure S18.** ^1^H-^13^C HSQC NMR spectrum of 4'-*O*-methylisoscutellarein 7-*O*-[6'''-*O*-acetyl-β-D-allopyranosyl-(1→2)-β-D-glucopyranoside] (**38**) (pyridine-*d*_5_, 600/151 MHz)

**Figure S19.** ^1^H-^13^C HMBC NMR spectrum of 4'-*O*-methylisoscutellarein 7-*O*-[6'''-*O*-acetyl-β-D-allopyranosyl-(1→2)-β-D-glucopyranoside] (**38**) (pyridine-*d*_5_, 600/151 MHz)

**Figure S20.** ^1^H NMR spectrum of 4'-*O*-methylisoscutellarein 7-*O*-[6'''-*O*-acetyl-β-D-allopyranosyl-(1→2)-β-D-glucopyranoside] (**38**) (DMSO-*d*_6_, 400 MHz)

**Figure S21.** ^13^C NMR spectrum of 4'-*O*-methylisoscutellarein 7-*O*-[6'''-*O*-acetyl-β-D-allopyranosyl-(1→2)-β-D-glucopyranoside] (**38**) (DMSO-*d*_6_, 101 MHz)

**Figure S22.** ^1^H-^1^H COSY NMR spectrum of 4'-*O*-methylisoscutellarein 7-*O*-[6'''-*O*-acetyl-β-D-allopyranosyl-(1→2)-β-D-glucopyranoside] (**38**) (DMSO-*d*_6_, 400/400 MHz)

**Figure S23.** ^1^H-^13^C HSQC NMR spectrum of 4'-*O*-methylisoscutellarein 7-*O*-[6'''-*O*-acetyl-β-D-allopyranosyl-(1→2)-β-D-glucopyranoside] (**38**) (DMSO-*d*_6_, 400/101 MHz)

**Figure S24.** ^1^H-^13^C HMBC NMR spectrum of 4'-*O*-methylisoscutellarein 7-*O*-[6'''-*O*-acetyl-β-D-allopyranosyl-(1→2)-β-D-glucopyranoside] (**38**) (DMSO-*d*_6_, 400/101 MHz)

**Figure S25.** ^1^H NMR spectrum of 4'-*O*-methylhypolaetin 7-*O*-[6'''-*O*-acetyl-β-D-allopyranosyl(1→2)-β-D-glucopyranoside] (**28**) (pyridine-*d*_5_, 600 MHz)

**Figure S26.** ^13^C NMR spectrum of 4'-*O*-methylhypolaetin 7-*O*-[6'''-*O*-acetyl-β-D-allopyranosyl(1→2)-β-D-glucopyranoside] (**28**) (pyridine-*d*_5_, 600 MHz)

**Figure S27.** ^1^H-^1^H COSY NMR spectrum of 4'-*O*-methylhypolaetin 7-*O*-[6'''-*O*-acetyl-β-D-allopyranosyl(1→2)-β-D-glucopyranoside] (**28**) (pyridine-*d*_5_, 600/600 MHz)

**Figure S28.** ^1^H-^13^C HSQC NMR spectrum of 4'-*O*-methylhypolaetin 7-*O*-[6'''-*O*-acetyl-β-D-allopyranosyl(1→2)-β-D-glucopyranoside] (**28**) (pyridine-*d*_5_, 600/151 MHz)

**Figure S29.** ^1^H-^13^C HMBC NMR spectrum of 4'-*O*-methylhypolaetin 7-*O*-[6'''-*O*-acetyl-β-D-allopyranosyl(1→2)-β-D-glucopyranoside] (**28**) (pyridine-*d*_5_, 600/151 MHz)

**Figure S30.** *Stachys hissarica* Regel (N2016023) were collected from the Chimgan mountains (41°31'52.0"N 70°01'54.0"E) (Tashkent region, Uzbekistan)
